# Hope

**DOI:** 10.1017/S1478951525100849

**Published:** 2025-10-01

**Authors:** William Breitbart

**Affiliations:** Department of Psychiatry and Behavioral Sciences, Memorial Sloan-Kettering Cancer Center, New York, NY, USA

Hope

is

the Courage

to Create

an

Uncertain Future

-Bill Breitbart MD-

